# Atezolizumab Plus Bevacizumab Combination Therapy in Unresectable Hepatocellular Carcinoma: An Institutional Experience

**DOI:** 10.3390/biomedicines13122844

**Published:** 2025-11-21

**Authors:** Abdullah Esmail, Yazan Hamadneh, Bayan Khasawneh, Maryam Al-Rawi, Ebtesam Al-Najjar, Vikram Dhillon, Ahmad Alhaj, Yaser Rayyan, Maen Abdelrahim

**Affiliations:** 1 Section of GI Oncology, Department of Medicine, Houston Methodist Cancer Center, Houston, TX 77030, USA; aesmail@houstonmethodist.org (A.E.);; 2 Department of Medicine, Weill Cornell Medical College, New York, NY 10065, USA; 3 Faculty of Medicine, The University of Jordan, Amman 11942, Jordan

**Keywords:** atezolizumab, bevacizumab, hepatocellular carcinoma, unresectable HCC, real-world evidence, overall survival, progression-free survival, locoregional therapy, Child–Pugh, immune checkpoint inhibitors

## Abstract

**Background:** Hepatocellular carcinoma (HCC) is a leading cause of cancer-related mortality. Atezolizumab plus bevacizumab (Atezo/Bev) has emerged as a first-line therapy for unresectable HCC (uHCC), improving overall and progression-free survival (OS, overall survival and PFS, progression-free survival) in IMbrave150. This study evaluates the real-world efficacy and safety of Atezo/Bev in uHCC. **Methods:** A retrospective analysis was performed on 87 patients (median age 68 years) treated with Atezo/Bev at Houston Methodist Hospital between January 2020 and June 2023. Demographics, treatment patterns, radiological response, OS, PFS, and toxicities were reviewed. Atezo/Bev was administered per FDA guidelines (atezolizumab 1200 mg plus bevacizumab 15 mg/kg every 3 weeks). **Results:** Of 87 patients, 78% were male, 71% White, and 70% had BCLC stage C disease. Most (60%) had Child–Pugh class A liver function, and 62% had viral hepatitis. Median OS was 15.1 months (95% CI: 10.57–25.97) and PFS was 9.1 months (95% CI: 7.4–21.07). Objective response rate was 31.3% (CR 7.2%, PR 25%, SD 52%, PD 16%). OS was longer in CP A versus CP B patients (21.2 vs. 5.2 months, *p* < 0.001) and in those receiving post-Atezo/Bev locoregional therapy (21.2 vs. 10.4 months, *p* = 0.043). Discontinuation due to toxicity occurred in 14%, mainly gastrointestinal bleeding and fatigue. **Conclusions:** Atezo/Bev demonstrated favorable real-world efficacy and manageable toxicity in uHCC, particularly in patients with preserved liver function or multimodal therapy.

## 1. Introduction

Hepatocellular carcinoma (HCC) is the most common primary liver cancer and a major cause of cancer-related deaths worldwide [1]. It ranks third among the deadliest cancers worldwide and the seventh in the United States (US) [2]. Primary predisposing risk factors linked to HCC include chronic viral hepatitis due to hepatitis B virus (HBV) or hepatitis C virus (HCV), liver cirrhosis, Diabetes Mellitus (DM), alcohol, non-alcoholic steatohepatitis (NASH), and non-alcoholic fatty liver disease (NAFLD) [3,4,5,6]. A recent Surveillance Epidemiology and End Results (SEER) database study showed an increase in liver cancer incidence and mortality rates in the US [7]. Incidence was higher in men, possibly due to a greater prevalence of the aforementioned risk factors among them [8]. Additionally, mortality rates were three times higher in men than in women. Common presenting symptoms can include abdominal pain, fatigue, weight loss, jaundice, and ascites. However, HCC is usually asymptomatic in its early stages, thus leading to diagnosis at advanced stages which deems patients ineligible for curative surgery and offers limited effective therapeutic options [9].

Early detection, done by screening, is recommended for high-risk patients and is crucial for achieving better outcomes. The use of non-invasive biomarkers like Alpha-Fetoprotein (AFP) and imaging modalities such as ultrasound, Computed Tomography (CT), or Magnetic Resonance Imaging (MRI) were investigated as methods for early detection of HCC. A combined approach of these methods has raised the sensitivity and specificity of HCC diagnosis [10,11,12]. Furthermore, Artificial Intelligence (AI) and machine learning were utilized to screen for tumor recurrence based on previous MRI scans in patients with early stages of HCC [13].

Recent studies and research regarding systemic and Locoregional Therapies (LRT) have led to alterations in HCC treatment guidelines by implementing more effective therapies which allowed some patients with unresectable diseases to downstage tumors and undergo surgical resection [14].

Management of the disease is based on the stage of HCC and the liver function. Surgical options including resection and Orthotopic Liver Transplant (OLT), or local ablative therapies, like Radiofrequency Ablation (RFA) or Microwave Ablation (MWA) are reserved for patients with early-stage HCC. While for intermediate-stage HCC, or as bridging therapy prior to surgery or OLT, LRT such as yttrium-90 radioembolization (Y-90), stereotactic body radiation therapy (SBRT), and transarterial chemoembolization (TACE) are utilized [15,16]. On the other hand, systemic therapies, such as targeted therapy, immunotherapy, and chemotherapy, are recommended in advanced and unresectable HCC [17].

Recent studies showed that Immune Checkpoint Inhibitors (ICPIs) induced an appreciable improvement in tumor response while carrying a lower risk profile, leading to a revolution in the treatment of HCC. IMbrave150 and other large phase III trials which tested the efficacy and safety of using atezolizumab and bevacizumab (Atezo/Bev) versus sorafenib in patients with unresectable HCC. Results showed a statistically significant and clinically meaningful improvement in the Overall Survival (OS) and Progression-free Survival (PFS) among patients treated with Atezo/Bev compared to sorafenib. After a median of 8.6 months of follow-up, Atezo/Bev were preferred as frontline treatments for HCC and were approved by the Food and Drug Administration (FDA) as of May 2020 [18]. The latest update of the IMbrave150 study showed that Atezo/Bev had a median OS of 19.2 months compared to sorafenib with a median OS of 13.4 months, which maintained the clinically improved treatment benefit [19].

Atezolizumab is an ICPI that targets the proteins responsible for regulating the immune response, known as immune checkpoints. It antagonizes the Programmed cell death protein 1 (PD-1) receptor on T-cells and the Programmed death-ligand 1 (PD-L1) protein on tumor cells leading to enhanced ability of T-cells to eliminate tumor cells and creating a robust immune response against the tumor. On the contrary, bevacizumab serves as a monoclonal antibody against the Vascular Endothelial Growth Factor (VEGF) protein, which is a potent angiogenic factor produced by many cells to stimulate the formation of blood vessels, thus impeding tumor growth and nourishment by cutting down tumor’s supply of oxygen and nutrients. This approach provides a synergistic therapeutic effect and has the potential to greatly improve patient outcomes in the treatment of HCC [20,21,22].

There is limited real-world evidence on patient characteristics, treatment patterns, and outcomes among patients with HCC treated with Atezo/Bev as well as durvalumab plus tremelimumab (Durva/Treme) and other treatments. The main objective of this study is to evaluate further the efficacy of implementing atezolizumab in combination with bevacizumab for the treatment of unresectable HCC.

## 2. Methods

### Study Design and Participants

We retrospectively conducted data for 87 HCC patients with a median age of 68 who received Atezo/Bev as a first or later line of treatment for HCC at Houston Methodist Neal Cancer Center. Eligibility criteria included patients with a confirmed HCC diagnosis who received Atezo/Bev combination therapy and who were not diagnosed with any other primary tumor within 5 years of treatment initiation. Patients who received any other systemic therapy, LRT, or both, were eligible for inclusion. It was ensured that all included patients had enough data to report and that they were not lost to follow-up. Data were collected for patients treated with Atezo/Bev between January 2020 and June 2023. HCC etiologies were classified as either viral (HBV, HVC) or non-viral causes. Laboratory studies including AFP, Serum Albumin, Bilirubin, Aspartate transaminase (AST), Alanine transaminase (ALT), Alkaline Phosphatase (ALP), Prothrombin time (PT), International Normalized Ratio (INR) were collected at the time of treatment initiation with atezolizumab and bevacizumab. Comorbidities including DM, Hypertension (HTN), chronic kidney disease (CKD), hemochromatosis, and disease-related complications such as Encephalopathy, Esophageal varices, and Ascites were documented. Dates and findings of Esophagogastroduodenoscopy (EGD) procedures were collected at the time of treatment initiation to determine treatment eligibility and to check for the presence of varices that rendered patients ineligible for treatment. Several classifications were utilized to determine the patient’s status and disease stage at the time of treatment initiation with Atezo/Bev, such as Eastern Cooperative Oncology Group Performance Status (ECOG PS), Barcelona Clinic Liver Cancer (BCLC), and Child-Pugh (CP) staging system. The study documented the reasons for treatment discontinuation as either related to treatment toxicity, which included signs and symptoms like Upper GI Bleeding (UGIB), abnormal electrolytes, elevated liver enzymes, etc., or other reasons related to insurance coverage or patient’s personal preferences. Previous treatment with other systemic therapy, LRT, or surgery were documented before and after treatment with Atezo/Bev. Subjects were followed up and results were documented based on radiographic imaging which included CT, MRI, and Positron Emission Tomography–Computed Tomography (PET-CT). The best response of treatment was classified as either Complete response (CR), Partial response (PR), Stable Disease (SD), or Progression of Disease (PD). Treatment was administered based on the FDA-recommended atezolizumab dose of 1200 mg, followed by 15 mg/kg bevacizumab on the same day every 3 weeks. The primary endpoints were the OS and PFS.

## 3. Results

### 3.1. Patient Characteristics

A total of 87 patients were included in this retrospective cohort, of whom 70 received Atezo/Bev as first-line therapy and 17 as later-line therapy. The mean age at the time treatment began was 68 years. A majority of the patients were male (78%) and nearly all (99%) identified as Hispanic or Latino in terms of ethnicity. Within this group, patients self-identified racially as White (71%), Black (14%), Asian (13%), and American Indian or Alaska Native (1.2%).

The majority of patients had an ECOG PS of 0–1 (88%) and ECOG PS ≥ 2 (11.1%). Prior to treatment initiation, cirrhosis (77%) and HTN (66%) as comorbid conditions. Overall, 62% of patients had viral hepatitis (HBV or HCV). Viral hepatitis, primarily HCV, was the leading underlying etiology of HCC (59%), followed by non-viral causes (40%). Liver function was categorized as Child–Pugh class A in 60% of patients, B in 34%, and C in 1.1%. Most patients (70%) were classified as BCLC stage C.

Among those with viral hepatitis, 43 (49%) were HCV-positive with a median infection duration of approximately 8 years; all achieved sustained virologic response (SVR) following direct-acting antiviral therapy, most commonly with sofosbuvir/velpatasvir or Vosevi. Eleven patients (13%) were HBV positive and received antiviral therapy (entecavir or tenofovir) prior to and during systemic treatment. Seventeen patients (20%) had a history of alcohol-related liver disease, while 16 (18%) had non-viral, non-alcoholic etiologies, including NASH and cryptogenic cirrhosis (Table 1) and (Tables S1–S6).

### 3.2. Treatment Patterns

Prior to starting Atezo/Bev, over half of patients had received LRT (58 patients, 67%), and 17 patients (20%) had received prior systemic therapy. Among patients who discontinued Atezo/Bev during the study period, the primary reason for discontinuation was disease progression or transition to hospice care (46 patients, 55%). Following Atezo/Bev treatment, a subset of patients (18 patients, 20%) went on to receive subsequent systemic therapy.

### 3.3. Baseline EGD Evaluation

At baseline, the majority of patients in the cohort had undergone at least one EGD, with the median time between the most recent procedure and initiation of Atezo/Bev being 1.1 months. Among those who had ≥1 EGD, procedures were primarily conducted to assess treatment eligibility for Atezo/Bev or as part of the routine diagnostic workup for HCC. Esophageal varices were identified in (26 patients, 33%) who underwent EGD, and of those, 27% received appropriate treatment (Table 2).

### 3.4. Efficacy

**Figure 1:**
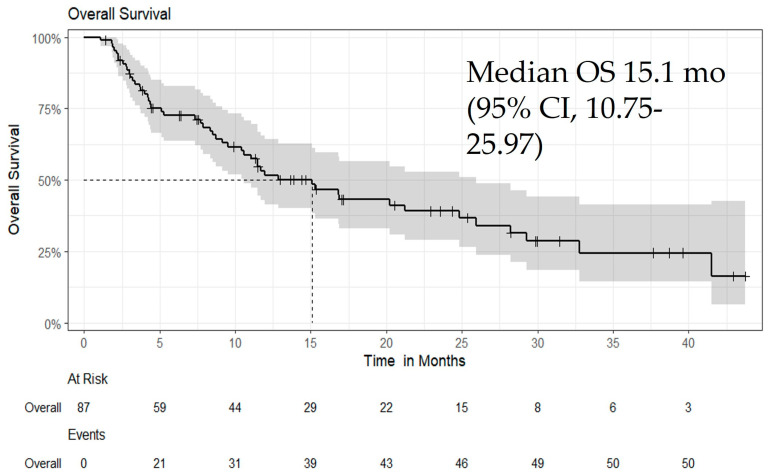
Kaplan–Meier curve for OS in all patients treated with Atezo/Bev, showing a median OS of 15.1 months (95% CI: 10.57–25.97; *p* = 0.06). This figure illustrates the Kaplan–Meier survival analysis for OS among patients who received Atezo/Bev as first-line or later-line therapy. Median OS was 15.1 months, with a trend toward improved survival (*p* = 0.06). Censored observations are indicated by tick marks.

**Figure 2:**
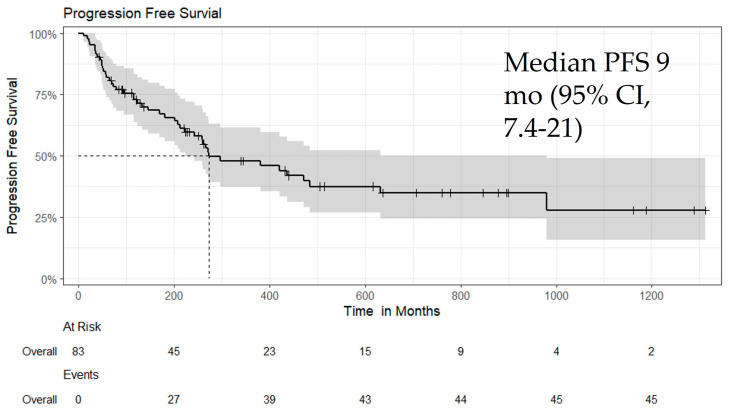
Kaplan–Meier curve for PFS in all patients treated with Atezo/Bev, showing a median PFS of 9.1 months (95% CI: 7.4–21.07; *p* = 0.05). This figure depicts the Kaplan–Meier analysis of progression-free survival among patients who received Atezo/Bev as first-line or later-line therapy. Median PFS was 9.1 months, with a significant trend toward prolonged disease control (*p* = 0.05). Censored data points are indicated by tick marks.

All patients received Atezo/Bev as first-line or later-line therapy. The median follow-up duration was 10 months (interquartile range [IQR], 4–20), and the median time to best response was 3.4 months (IQR, 1.9–7.6). On follow-up imaging, the best overall responses were CR in 7.2%, PR in 25%, SD in 52%, and PD in 16% of patients. The objective response rate (ORR) in the overall cohort was 31.3% (Table 3). Median OS was 15.1 months (95% CI: 10.57–25.97; *p* = 0.06) (Figure 1), and median PFS was 9.1 months (95% CI: 7.4–21.07; *p* = 0.05) (Figure 2). Additional subgroup survival analyses are presented in Supplementary Figures S1–S10.

### 3.5. Subgroup Analysis

OS varied significantly by race/ethnicity (*p* = 0.012) with Asian patients showing a longer median OS of 12.8 months (95% CI: 3.7, not reached) compared to others. Based on CP class, OS differed significantly between CP A and CP B patients (*p* < 0.001). Median OS was notably longer in CP A patients at 21.2 months (95% CI: 15.1–not reached) compared with 5.2 months (95% CI: 3.2–11.7) in CP B patients, reflecting the expected survival advantage in patients with preserved liver function. Patients with viral hepatitis-particularly those with HCV-tended to have a more favorable prognosis, with a median OS of 15.3 months (95% CI: 11.4, not reached), compared to 11.4 months (95% CI: 8.33, not reached) in patients without viral-related HCC; however, this difference was not statistically significant (*p* = 0.8).

All patients in this study received Atezo/Bev treatment, with 17 receiving it as a later-line therapy. Patients receiving first-line therapy had a median OS of 16.83 months (95% CI: 11.97, not reached), compared to 11.47 months (8.7, not reached), in those treated in later lines, although this difference was not statistically significant (*p* = 0.4).

LRT following Atezo/Bev was associated with significantly improved OS (21.20 vs. 10.40 months, *p* = 0.043). Additionally, patients who underwent surgery after Atezo/Bev had a median OS of 26.98 months, compared to 11.70, respectively, in those who did not undergo surgery. These results suggest improved outcomes with Atezo/Bev in patients with well-preserved liver function and when used as part of a multimodal treatment strategy.

PFS also varied significantly by race/ethnicity (*p* = 0.018) with white patients showing a longer median PFS of 9.1 months (95% CI: 7.4, not reached) compared to others. Based on CP class, PFS differed significantly between CP A and CP B patients (*p* = 0.013), with the median PFS longer in CP A patients (14.57 [95% CI: 9.1, not reached]) vs. CP B patients (6 months [95% CI: 2.6, 21.07]). Patients with viral hepatitis-particularly those with HCV tended to have a more favorable prognosis, with a median OS of 16.17 months (95% CI: 5.6, not reached), compared to 9.1 months (95% CI: 8.1, not reached) in patients without viral-related HCC; however, this difference was not statistically significant (*p* = 0.2).

Median PFS was 14 months (95% CI: 8.6–not reached) in first-line therapy and 6 months (95% CI: 3.1—not reached) in later-line therapy (*p* = 0.14). PFS was comparable between patients who did and did not receive post-treatment LRT (9.10 vs. 9.87 months, *p* = 0.8). Patients who underwent surgery after Atezo/Bev had a longer median PFS (16.17 vs. 9.10 months, *p* = 0.6), though this difference was not statistically significant. (see Supplementary Tables S1–S6 for additional subgroup details).

### 3.6. Safety

The discontinuation rate of Atezo/Bev due to toxicity was 14%. Reported reasons for discontinuation included upper GI bleeding (3.4%), poor appetite and fatigue (3.4%), abnormal electrolytes (2%), elevated thyroid-stimulating hormone (TSH) (1%), grade 3 hepatitis (1%), elevated liver enzymes (1%), and intracerebral hemorrhage (1%) (Table 4).

Safety events occurred at different time points during treatment, ranging from early to later cycles. Among the 12 patients who experienced significant adverse events, 4 died due to disease progression, and 3 required a change in treatment setting. The remaining patients were managed symptomatically and continued Atezo/Bev as tolerated.

## 4. Discussion

This real-world retrospective analysis assessed the effectiveness of Atezo/Bev in patients with uHCC treated at a single institution. The cohort provides valuable clinical data offering insights into the effectiveness of current and future therapies.

Compared to IMbrave 150, the population in our study was older (mean age 68 vs. 64 years), with broader inclusion of individuals who would have been ineligible for trial enrollment. Notably, 35% of patients in our cohort had CP B or C liver function, and 11% had an ECOG PS ≥ 2 both of which were exclusion criteria in the IMbrave 150 trial. In contrast IMbrave 150 enrolled only CP A patient with ECOG PS 0 -1. Our cohort also included a more racially and ethnically diverse population and a higher proportion of patients with viral hepatitis, particularly HCV. While HBV was the predominant cause in the IMbrave 150 trial.

Despite this difference, the clinical outcome in our real-world was consistent or modestly better than those reported in the IMbrave 150 trial. We observed a median OS of 15.1 months and median PFS of 9.1 months, compared to 19.2 months and 6.9 months, respectively, in IMbrave 150. Additionally, the ORR was also slightly higher in our study at 31.3% to 27% in IMbrave 150 based on independent review facility (IRF)-assessed RECIST 1.1 criteria [18].

Importantly, emphasizing patients with preserved liver function CP A, we found that OS estimates in our cohort were significantly better than those reported in the IMbrave 150 trial, with CP A patients demonstrating a median OS of 21.2 months.

Liver function is a key determinant of prognosis and treatment response in HCC. In our cohort, patients with preserved hepatic reserve (Child–Pugh A) achieved markedly superior outcomes compared to those with Child–Pugh B, consistent with findings from clinical and real-world studies [1,2,3,4,5]. The IMbrave150 trial enrolled only Child–Pugh A patients, reaffirming the importance of adequate hepatic function for Atezo/Bev efficacy and tolerability [1,2]. Real-world evidence indicates that while Atezo/Bev can be administered in selected Child–Pugh B patients, these individuals experience lower survival rates and higher toxicity, largely due to impaired hepatic metabolism and immune dysfunction [3,4,5]. Preserving hepatic function through optimal cirrhosis management and early intervention remains crucial to maximizing treatment benefit [6,7,8]. Our findings align with growing data that maintaining Child–Pugh A status correlates with prolonged survival and improved treatment tolerance in Atezo/Bev-treated patients [8,9]. Our findings align with a growing body international real-world data demonstrating the effectiveness of Atezo/Bev across divers populations. For instance, D’Alessio et al. reported a median OS of 14.9 months and PFS of 6.8 months in multicenter study spanning seven countries. In France, Allaire et al. observed median OS of 23.7 months in large, based cohort Similar outcomes were reported in Germany and Austria, where Himmelsbach et al. and De Castro et al. found median PFS ranging from 5.1 to 8.7 months depending on patient characteristics. Fulgenzi et al. also reported a PFS of 6.9 months in patients with favorable baseline profiles across tertiary cancer in the U.S., Europe, and Asia [23]. In the U.S., Abdelrahim et al. conducted a multicenter study involving five major institutions, showing a median OS of 14.4 months and PFS of 6.8 months overall, with improved outcomes in trial-like patients (OS 19.5 months, PFS 8.8 months) [24].

In our cohort, patients with non-viral HCC appeared to have inferior outcomes than those with viral-related HCC, although the difference did not reach statistical significance. A meta-analysis of three randomized trials evaluating ICPIs (IMbrave 150, CheckMate 459, and KEYNOTE-240) demonstrated that OS were better in patients with HBV or HCV related to HCC, whereas those with non-viral HCC did not experience a significant benefit [25]. Inada et al. and other researchers have reported findings that support the weak immunotherapy response observed in NASH patients. Recently, Pfister et al. published widely discuses evidence showing that immunotherapy is less effective in patient with HCC associated with NASH compared to other HCC patients. This reduced effectiveness is linked to the presence of unique resident-like activated CD8+ T cells in NASH related HCC [26].

Subgroup analysis in our cohort highlighted meaningful difference in outcomes. Patients with preserved liver function CP A had significantly longer OS compared to those with CP B, emphasizing the critical role of baseline hepatic reserve in guiding treatment decision with Atezo/Bev. Additionally, patients with viral-related HCC, particularly HCV, tended to have more favorable outcomes than those with non-viral etiologies consistent with prior reports suggesting that immunotherapy responses may be weaker in non-viral or NASH-related HCC. These findings underscore the importance of considering both liver function and underlying etiology when predicting prognosis and tailoring therapy in real-world practice.

Our cohort demonstrated a higher rate of esophageal varices detection (33%) and treatment (27%) compared to the IMbrave 150 trial (26% and 11%, respectively). This finding is consistent with a real-world cohort study published by Lee et al., which reported varices detection and treatment rates of 41% and 19%, respectively. This likely reflects the inclusion of patients with more advanced liver disease and cirrhosis in real-world practice [27]. Despite implementing a selective approach in which most patients underwent baseline EGD, the incidence of GI bleeding in our study (3%) was lower to rates reported in the IMbrave 150 trial (7%) and the aforementioned cohort study (5%).

Moreover, our findings are in line with bleeding rates observed in other clinical trials involving anti-VEGF agents combined with ICPIs. In the ORIENT-32 trial, treatment with sintilmab plus IBI305 (a bevacizumab biosimilar) resulted in a 5% GI bleeding rate. The COSMIC-312 trial, evaluating atezolizumab and cabozantinib, reported a rate of less than 1%. Similarly, the LEAP-002 trial mandated a baseline EGD within three months prior to initiating lenvatinib and pembrolizumab and observed an upper GI bleeding incidence of less than 1%. In the CARES-310 trial, the combination of rivoceranib and camrelizumab was associated with a 3% incidence of upper GI bleeding [27].

In addition to our real-world outcomes, it is important to situate Atezo/Bev within the border therapeutic landscape of advanced HCC. Combination immunotherapy regimens in HCC have generally demonstrated superior response rate and longer duration of response when anti- PD-L1 inhibitor with anti-VEGF antibody or anti-CTLA-4 therapies. Mechanistically, anti-PD-L1 agents such as atezolizumab prevent PD-L1 from binding to both PD-1 and B7-1, potentially enhancing T-cell activation, whereas anti-PD-1 agent block PD-1 linked by PD-L1 and PD-L2. Bevacizumab, by inhibiting VEG, not only suppress angiogenesis but also reduces VEGF-mediated immunosuppression, promoting T-cell infiltration and synergizing with checkpoint inhibition. This dual mechanism differs fundamentally from TKIs such a sorafenib or lenvatinib, which primarily inhibit tumor proliferation and angiogenesis without directly stimulating anti-tumor immunity, and whose safety profiles differ, with TKIs more commonly associated with palmer-planter erythrodysesthesia and bevacizumab with HTN or proteinuria. Collectively, these mechanistic and safety considerations help contextualize the durable responses and tolerability observed with Atezo/Bev in our real-world cohort.

In our cohort, patients who received LRT after Atezo/Bev demonstrated a significantly longer OS compared to those who did not (21.20 vs. 10.40 months, *p* = 0.043). A key challenge in managing patients with SD during Atezo/Bev therapy is how to improve their prognosis, as they may not respond well to immunotherapy alone. The suggestion is that combining Atezo/Bev with LRT can enhance treatment effectiveness. This strategy may create an environment in which the therapeutic effect can be maintained and further prolong the prognosis.

Supporting this observation, a study by Hosui et al. demonstrated a similar survival benefit of adding LRT in HCC patients with SD following initial treatment with Atezo/Bev. In contrast, no such benefit was seen in patients treated with lenvatinib [28]. The authors propose that the unique immunological mechanism of action of atezolizumab. As an ICPI, atezolizumab reactivates cytotoxic T cells by blocking the PD-L1 pathway, thereby preventing immune escape from tumor cells. LRT like TACE or RFA can further enhance this effect by inducing immunogenic cell death and increasing tumor antigen release. This synergistic interaction is not expected with agents like lenvatinib, which lack immunomodulatory effects. Thus, the combination of Atezo/Bev with LRT may help sustain or deepen the treatment response in patients with otherwise SD [28].

This study has several limitations that should be acknowledged. As a retrospective, single-center analysis, it may be subject to selection bias, as the included patients may not fully represent the broader population of individuals with unresectable HCC.

The observational nature of the study also limits our ability to draw causality, and unmeasured confounding factors could have influenced the outcomes. Additionally, variations in clinical practice, follow-up schedules, and supportive care may have affected the results. Furthermore, patient quality-of-life and cost-effectiveness analyses were not available in this retrospective study and represent important areas for future research.

Despite these limitations, the finding provides valuable insights into the real-world effectiveness of Atezo/Bev in a diverse patient population.

## 5. Conclusions

This real-world study reinforces the clinical value of Atezo/Bev for uHCC, particularly in patients often excluded from pivotal trials due to poorer liver function or performance status. The outcomes observed in our cohort not only mirror but, in some subgroups, exceed those seen in IMbrave150, especially among patients with Child–Pugh A liver function. Furthermore, our findings suggest that the addition of LRT may enhance survival outcomes in patients with stable disease under Atezo/Bev treatment. These insights contribute to the evolving treatment paradigm and support a more personalized approach in managing advanced HCC in routine clinical practice.

However, these results should be interpreted with caution due to the retrospective and single-center nature of the study, the relatively small sample size, and the heterogeneity of the patient population, including both first- and later-line therapy groups. While ongoing prospective trials continue to explore Atezo/Bev and its combinations, additional real-world data and multicenter analyses are needed to confirm these observations and refine patient selection strategies in advanced HCC.

## Figures and Tables

**Table 1:** Baseline Demographics and Clinical Characteristics of the Study Cohort (N = 87).

Characteristics	n (%) or Median Range
**Age at Initiation (years)**	Median (Q1–Q3): 68 (61–73); Range: 29–91
**Sex**	
Female	19 (22%)
Male	68 (78%)
**Ethnicity**	
Hispanic or Latino	86 (99%)
Unknown	1 (1%)
**Race**	
American Indian or Alaska Native	1 (1.2%)
Asian	11 (13%)
Black	12 (14%)
White	60 (71%)
Unknown	3 (3%)
**Vital Status**	
Alive	36 (41%)
Deceased	51 (59%)
**Disease Progression**	
No Progression	38 (45%)
Progression	46 (55%)
**Child–Pugh Score**	
5	24 (28%)
6	28 (33%)
7	13 (15%)
8	12 (14%)
9	7 (8.2%)
10	1 (1.1%)
**Child–Pugh Class**	A: 52 (60%); B: 34 (39%); C: 1 (1.1%)
**BCLC Stage**	A: 8 (9.2%); B: 18 (21%); C: 61 (70%)
**ECOG Performance Status**	0: 21 (24%); 1: 56 (64%); 2: 9 (10%); 3: 1 (1.1%)

Values are presented as n (%) unless otherwise indicated. Abbreviations: ECOG, Eastern Cooperative Oncology Group; BCLC, Barcelona Clinic Liver Cancer; CP, Child–Pugh.

**Table 2:** Endoscopic Findings and Management of Varices Among the Study Cohort.

	Total (N = 87)
Category	EGD Performed (N = 79)
**Patient with Varices**	26
**Treatment for Varices**	7
Band ligation	6
Beta-blockers	1
**No treatment required**	13

Values are presented as number of patients unless otherwise indicated. Abbreviation: EGD, esophagogastroduodenoscopy.

**Table 3:** Best Overall Response and Treatment Duration Among Patients Treated with Atezo/Bev (N = 87).

Characteristic	n (%) or Median (Range)
**Best Overall Response**	
Complete response	6 (7.2%)
Disease progression	13 (16%)
Partial response	21 (25%)
Stable disease	43 (52%)
**Follow-up Time (months)**	
Median (Q1, Q3)	10 (Total N = 44)
Min, Max	1, 44
**Time to Best Response**	
Median (Q1, Q3)	3.4 (1.9, 7.6)
Min, Max	0.1, 27.7

This table summarizes the best overall response, follow-up duration, and time to best response among all patients who received with Atezo/Bev. Responses were assessed by imaging according to clinician evaluation. Median follow-up was 10 months (Q1–Q3, 4–20), and the median time to best response was 3.4 months (Q1–Q3, 1.9–7.6).

**Table 4:** Treatment-Related Adverse Events in Patients Treated with Atezolizumab Plus Bevacizumab (N = 87).

Adverse Event	Yes, n (%)	No, n (%)
**Any Atezo/Bev Toxicity**	12 (14%)	75 (86%)
**Abnormal Electrolytes**	2 (2.3%)	85 (98%)
**Upper GI Bleeding**	3 (3.4%)	84 (97%)
**Intracerebral Hemorrhage**	1 (1.1%)	86 (99%)
**Elevated TSH**	1 (1.1%)	86 (99%)
**Grade 3 Hepatitis**	1 (1.1%)	86 (99%)
**Elevated Liver Enzymes**	1 (1.1%)	86 (99%)
**Poor Appetite & Fatigue**	3 (3.4%)	84 (97%)

Values are presented as number of patients (percentage). Abbreviations: Atezo/Bev, atezolizumab plus bevacizumab; GI, gastrointestinal; TSH, thyroid-stimulating hormone.

## Data Availability

The data supporting the findings of this retrospective analysis titled “Atezolizumab plus Bevacizumab Combination Therapy in Unresectable Hepatocellular Carcinoma: An Institutional Experience” are available upon request from the corresponding author, Maen Abdelrahim.

## Supplementary Materials

The following supporting information can be downloaded at: https://www.mdpi.com/article/10.3390/biomedicines13122844/s1; Table S1: Demographics; Table S2: Treatment response and HCC primary cause; Table S3: Additional treatments and Labs; Table S4: Comorbidities; Table S5: Total Toxicity and discontinuation Causes; Table S6: OS and PFS all table; Figure S1: Kaplan–Meier OS curves stratified by Child–Pugh score; Figure S2: Kaplan–Meier PFS curves stratified by Child–Pugh score; Figure S3: Kaplan–Meier OS curves stratified by sex (male vs. female); Figure S4: Kaplan–Meier PFS curves stratified by sex (male vs. female); Figure S5: Kaplan–Meier PFS curves stratified by race; Figure S6: Kaplan–Meier OS curves stratified by race; Figure S7: OS by receipt of locoregional therapy (LRT); Figure S8: PFS by receipt of locoregional therapy (LRT); Figure S9: OS in Child–Pugh Class A patients by line of therapy; Figure S10: OS in Child–Pugh Class B patients by line of therapy.

## Abbreviations

**Abbreviation**	**Full Term**
HCC	Hepatocellular carcinoma
uHCC	unresectable Hepatocellular carcinoma
Atezo/Bev	Atezolizumab plus bevacizumab
OS	overall survival
PFS	progression-free survival
EGD	esophagogastroduodenoscopy
BCLC	Barcelona Clinic Liver Cancer
CP	Child-Pugh
US	United States
HBV	hepatitis B virus
HCV	hepatitis C virus
DM	Diabetes Mellitus
NASH	alcohol, non-alcoholic steatohepatitis
NAFLD	non-alcoholic fatty liver disease
SEER	Surveillance Epidemiology and End Results
AFP	Alpha-Fetoprotein
CT	Computed Tomography
MRI	Magnetic Resonance Imaging
AI	Artificial Intelligence
LRT	Locoregional Therapies
OLT	Orthotopic Liver Transplant
RFA	Radiofrequency Ablation
MWA	Microwave Ablation
Y-90	yttrium-90 radioembolization
SBRT	stereotactic body radiation therapy
TACE	and transarterial chemoembolization
ICPIs	Immune Checkpoint Inhibitors
FDA	Food and Drug Administration
PD-1	Programmed cell death protein 1
PD-L1	Programmed death-ligand 1
VEGF	Vascular Endothelial Growth Factor
Durva/Treme	durvalumab plus tremelimumab
AST	Aspartate transaminase
ALT	Alanine transaminase
INR	International Normalized Ratio
PT	Prothrombin time
ALP	Alkaline Phosphatase
HTN	Hypertension
CKD	chronic kidney disease
ECOG PS	Eastern Cooperative Oncology Group Performance Status
UGIB	Upper GI Bleeding
PET-CT	Positron Emission Tomography–Computed Tomography
CR	Complete response
PR	Partial response
SD	Stable Disease
PD	Progression of Disease
ORR	objective response rate
TSH	thyroid-stimulating hormone

## References

[1] Asafo-Agyei K.O., Samant H. (2024). Hepatocellular Carcinoma. StatPearls.

[2] Sung H., Ferlay J., Siegel R.L., Laversanne M., Soerjomataram I., Jemal A., Bray F. (2021). Global Cancer Statistics 2020: GLOBOCAN Estimates of Incidence and Mortality Worldwide for 36 Cancers in 185 Countries. CA Cancer J. Clin..

[3] Gomaa A.I., A Khan S., Toledano M.B., Waked I., Taylor-Robinson S.D. (2008). Hepatocellular carcinoma: Epidemiology, risk factors and pathogenesis. World J. Gastroenterol..

[4] Suresh D., Srinivas A.N., Kumar D.P. (2020). Etiology of Hepatocellular Carcinoma: Special Focus on Fatty Liver Disease. Front. Oncol..

[5] Llovet J.M., Kelley R.K., Villanueva A., Singal A.G., Pikarsky E., Roayaie S., Lencioni R., Koike K., Zucman-Rossi J., Finn R.S. (2021). Hepatocellular carcinoma. Nat. Rev. Dis. Primers.

[6] Mak L.-Y., Cruz-Ramón V., Chinchilla-López P., Torres H.A., LoConte N.K., Rice J.P., Foxhall L.E., Sturgis E.M., Merrill J.K., Bailey H.H. (2018). Global Epidemiology, Prevention, and Management of Hepatocellular Carcinoma. Am. Soc. Clin. Oncol. Educ. Book..

[7] Howlader N., Noone A.M., Krapcho M., Miller D., Brest A., Yu M., Ruhl J., Tatalovich Z., Mariotto A., Lewis D.R. (2020). SEER Cancer Statistics Review, 1975–2017.

[8] Kulik L., El-Serag H.B. (2019). Epidemiology and Management of Hepatocellular Carcinoma. Gastroenterology.

[9] Abboud Y., Ismail M., Khan H., Medina-Morales E., Alsakarneh S., Jaber F., Pyrsopoulos N.T. (2024). Hepatocellular Carcinoma Incidence and Mortality in the USA by Sex, Age, and Race: A Nationwide Analysis of Two Decades. J. Clin. Transl. Hepatol..

[10] Yan Q., Sun Y.-S., An R., Liu F., Fang Q., Wang Z., Xu T., Chen L., Du J. (2023). Application and progress of the detection technologies in hepatocellular carcinoma. Genes. Dis..

[11] Yıldırım H., Kavgaci G., Chalabiyev E., Dizdar O. (2023). Advances in the Early Detection of Hepatobiliary Cancers. Cancers.

[12] Ayoub W.S., Steggerda J., Yang J.D., Kuo A., Sundaram V., Lu S.C. (2019). Current status of hepatocellular carcinoma detection: Screening strategies and novel biomarkers. Ther. Adv. Med. Oncol..

[13] Addissouky T.A., Sayed I.E., Ali M.M., Wang Y., Baz A.E., Khalil A.A., Elarabany N. (2024). Latest advances in hepatocellular carcinoma management and prevention through advanced technologies. Egypt. Liver J..

[14] Brown Z.J., Tsilimigras D.I., Ruff S.M., Mohseni A., Kamel I.R., Cloyd J.M., Pawlik T.M. (2023). Management of Hepatocellular Carcinoma: A Review. JAMA Surg..

[15] Lurje I., Czigany Z., Bednarsch J., Roderburg C., Isfort P., Neumann U.P., Lurje G. (2019). Treatment Strategies for Hepatocellular Carcinoma—A Multidisciplinary Approach. Int. J. Mol. Sci..

[16] Makary M.S., Khandpur U., Cloyd J.M., Mumtaz K., Dowell J.D. (2020). Locoregional Therapy Approaches for Hepatocellular Carcinoma: Recent Advances and Management Strategies. Cancers.

[17] He C., Zhang W., Zhao Y., Li J., Wang Y., Yao W., Wang N., Ding W., Wei X., Yang R. (2023). Preoperative prediction model for macrotrabecular-massive hepatocellular carcinoma based on contrast-enhanced CT and clinical characteristics: A retrospective study. Front. Oncol..

[18] Finn R.S., Qin S., Ikeda M., Galle P.R., Ducreux M., Kim T.-Y., Kudo M., Breder V., Merle P., Kaseb A.O. (2020). Atezolizumab plus Bevacizumab in Unresectable Hepatocellular Carcinoma. N. Engl. J. Med..

[19] Cheng A.L., Qin S., Ikeda M., Galle P.R., Ducreux M., Kim T.-Y., Lim H.Y., Kudo M., Breder V., Merle P. (2022). Updated efficacy and safety data from IMbrave150: Atezolizumab plus bevacizumab vs. sorafenib for unresectable hepatocellular carcinoma. J. Hepatol..

[20] Zhang L., Ding J., Li H.-Y., Wang Z.-H., Wu J. (2020). Immunotherapy for advanced hepatocellular carcinoma, where are we?. Biochim. Biophys Acta Rev. Cancer.

[21] Rizzo A., Brandi G. (2021). Biochemical predictors of response to immune checkpoint inhibitors in unresectable hepatocellular carcinoma. Cancer Treat. Res. Commun..

[22] Pinter M., Jain R.K., Duda D.G. (2021). The Current Landscape of Immune Checkpoint Blockade in Hepatocellular Carcinoma: A Review. JAMA Oncol..

[23] Cosgrove D., Tan R., Osterland A.J., Hernandez S., Ogale S., Mahrus S., Murphy J., Wilson T., Patton G., Loaiza-Bonilla A. (2025). Atezolizumab plus bevacizumab in patients with unresectable hepatocellular carcinoma: Real-world experience from a US Community Oncology Network. J. Hepatocell. Carcinoma.

[24] Abdelrahim M., Esmail A., Kim R.D., Arora S.P., Arshad J., Kournoutas I.A., O’donnell C.D., Totev T.I., Tan A., Mu F. (2025). Real-World Experiences Using Atezolizumab+ Bevacizumab for the Treatment of Unresectable Hepatocellular Carcinoma: A Multicenter Study. Cancers.

[25] Yiu D.C., Chan B.L.H., Wong A.C.F., Feng M.Y., Chan S.L. (2023). Real-world experiences of atezolizumab plus bevacizumab in patients with advanced hepatocellular carcinoma in Hong Kong. Liver Cancer Int..

[26] Himmelsbach V., Pinter M., Scheiner B., Venerito M., Sinner F., Zimpel C., Marquardt J.U., Trojan J., Waidmann O., Finkelmeier F. (2022). Efficacy and safety of atezolizumab and bevacizumab in the real-world treatment of advanced hepatocellular carcinoma: Experience from four tertiary centers. Cancers.

[27] Lee C.L., Freeman M., Burak K.W., Moffat G.T., O’donnell C.D.J., Ding P.Q., Lyubetska H., Meyers B.M., Gordon V., Kosyachkova E. (2024). Real-World Outcomes of Atezolizumab with Bevacizumab Treatment in Hepatocellular Carcinoma Patients: Effectiveness, Esophagogastroduodenoscopy Utilization and Bleeding Complications. Cancers.

[28] Hosui A., Hayata N., Kurahashi T., Namiki A., Okamoto A., Aochi K., Ashida M., Tanimoto T., Murai H., Ohnishi K. (2025). Efficacy of Adding Locoregional Therapy in ATZ/BEV-Treated Patients with Stable HCC. Cancers.

