# Supplementary material for: Atezolizumab Plus Bevacizumab Combination Therapy in Unresectable Hepatocellular Carcinoma: An Institutional Experience
Source: Biomedicines. 2025 Nov 21;13(12):2844. doi: 10.3390/biomedicines13122844 (PMC12730803; doi:10.3390/biomedicines13122844)
Supplement: Supplementary file 1 [file biomedicines-13-02844-s001.zip › biomedicines-3909102-supplementary.pdf]

Table S1. Demographics

Characteristic	N = 87[1]
**Age at Initiation**	
Median (Q1, Q3)	68 (61, 73)
Min, Max	29, 91
**Sex**	
F	19 (22%)
M	68 (78%)
**Ethnicity**	
Hispanic or Latino	86 (100%)
Unknown	1
**Race**	
American Indian or Alaska tive	1 (1.2%)
Asian	11 (13%)
Black	12 (14%)
White	60 (71%)
Unknown	3
**Vital Status (Alive/Deceased)**	
Alive	36 (41%)
Deceased	51 (59%)
**Disease Progression**	
No Progression	38 (45%)
Progression	46 (55%)
Unknown	3

[1] n (%)

Characteristic	N = 87[1]
**Child-Pugh Score**	
5	24 (28%)
6	28 (33%)
7	13 (15%)
8	12 (14%)
9	7 (8.2%)
10	1 (1.2%)
Unknown	2
**Child-Pugh Class**	
A	52 (60%)
B	34 (39%)
C	1 (1.1%)
**BCLC Stage**	
A	8 (9.2%)
B	18 (21%)
C	61 (70%)
**ECOG Performance Status**	
0	21 (24%)
1	56 (64%)
2	9 (10%)
3	1 (1.1%)

[1] n (%)

Table S2. Treatment response and HCC primary cause

Characteristic	N = 87[1]
**Best Overall Response**	
Complete response	6 (7.2%)
Disease progression	13 (16%)
Partial response	21 (25%)
Stable disease	43 (52%)
**Follow-up Time (months)**	
Median (Q1, Q3)	10 (4, 20)
Min, Max	1, 44
**Time to Best Response (months)**	
Median (Q1, Q3)	3.4 (1.9, 7.6)
Min, Max	0.1, 27.7
**Inpatient Admission (Yes/No)**	
No	28 (32%)
Yes	59 (68%)
**Time to inpatient addmission (months)**	
Median (Q1, Q3)	2.50 (0.60, 5.53)
Min, Max	-0.97, 14.83
**Time from EGD to Treatment (months)**	
Median (Q1, Q3)	1.1 (0.1, 2.2)
Min, Max	-3.6, 34.8
**Time from Diagnosis to EGD (months)**	
Median (Q1, Q3)	8 (3, 18)
Min, Max	0, 120

[1] n (%)

Characteristic	N = 87[1]
**HCC Primary cause**	
HBV	7 (9.7%)
HCV	36 (50%)
Non-viral	29 (40%)
Unknown	15

[1] n (%)

Characteristic	N = 87[1]
**Line of Therapy**	
First	70 (80%)
Second or More	17 (20%)
**Treatment Time (months)**	
Median (Q1, Q3)	5 (2, 12)

[1] n (%)

**ORR**	31.03%

Table S3. Additional treatments and Labs

Characteristic	N = 87[1]
**Prior Locoregional Therapy**	
No	29 (33%)
Yes	58 (67%)
**Prior Systemic Therapy**	
No	70 (80%)
Yes	17 (20%)
**Prior Surgery**	
No	85 (98%)
Yes	2 (2.3%)
**Locoregional Therapy After Atezo Bev**	
No	53 (61%)
Yes	34 (39%)
**Systemic Therapy After Atezo Bev**	
No	69 (79%)
Yes	18 (21%)
**Surgery After Atezo Bev**	
No	82 (94%)
Yes	5 (5.7%)

[1] n (%)

Characteristic	N = 87[1]
**Albumin**	3.50 (3.10, 3.90)
**ALT (IU/L)**	35 (24, 57)
**AST (IU/L)**	59 (39, 87)
**AFP (ng/ml)**	156 (16, 2,629)
**Abnormal AFP**	
Normal	17 (20%)
Abnormal	69 (80%)
**Bililrubin (mg/dL)**	0.70 (0.40, 1.20)
**INR Result**	1.20 (1.10, 1.30)
**PT (sec)**	14.50 (13.50, 16.10)

[1] Median (Q1, Q3); n (%)

Table S4. Comorbidities

Characteristic	N = 87[1]
**Varices**	
No	53 (67%)
Yes	26 (33%)
Unknown	8
**Treatment for Varices**	
Band ligation	6 (30%)
Beta- blockers	1 (5.0%)
No	13 (65%)
Unknown	67
**Cirrhosis**	
No	20 (23%)
Yes	67 (77%)
**Hepatitis C Virus (HCV)**	
No	44 (51%)
Yes	43 (49%)
**Hepatitis B Virus (HBV)**	
No	76 (87%)
Yes	11 (13%)
**Alcohol-related Liver Disease**	
No	70 (80%)
Yes	17 (20%)
**Fatty Liver Disease (FLD)**	
No	82 (94%)
Yes	5 (5.7%)
**Steatohepatitis (SH)**	
No	70 (80%)
Yes	17 (20%)
**Esophageal Varices**	
No	62 (71%)
Yes	25 (29%)
**Hepatic Encephalopathy**	
No	71 (82%)
Yes	16 (18%)
**Ascites**	
No	56 (64%)
Yes	31 (36%)
**Portal Hypertension**	
No	80 (92%)
Yes	7 (8.0%)
**Gastroesophageal Reflux Disease (GERD)**	
No	83 (95%)
Yes	4 (4.6%)
**Hyperlipidemia**	
No	78 (90%)
Yes	9 (10%)

**Hemochromatosis**	
No	83 (95%)
Yes	4 (4.6%)
**Hypothyroidism**	
No	84 (97%)
Yes	3 (3.4%)
**Chronic Kidney Disease (CKD)**	
No	83 (95%)
Yes	4 (4.6%)
**Diabetes Mellitus (DM)**	
No	46 (53%)
Yes	41 (47%)
**Hypertension (HTN)**	
No	30 (34%)
Yes	57 (66%)

[1] n (%)

Table S5. Total Toxicity and discontinuation Causes

Characteristic	N = 87[1]
**AtezoBev Toxicity (Yes/No)**	
No	75 (86%)
Yes	12 (14%)
**Abnormal Electrolytes**	
No	85 (98%)
Yes	2 (2.3%)
**Upper GI Bleeding**	
No	84 (97%)
Yes	3 (3.4%)
**Intracerebral Hemorrhage**	
No	86 (99%)
Yes	1 (1.1%)
**Poor Appetite & Fatigue**	
No	84 (97%)
Yes	3 (3.4%)
**Elevated TSH**	
No	86 (99%)
Yes	1 (1.1%)
**Grade 3 Hepatitis**	
No	86 (99%)
Yes	1 (1.1%)
**Elevated Liver Enzymes**	
No	86 (99%)
Yes	1 (1.1%)

Table S6. OS and PFS all table

Characteristic	OS (Months) 50% Percentile	OS (Months) p-value[1]	PFS (Months) 50% Percentile	PFS (Months) p-value[1]
**Overall**	15.10 (10.57, 25.97)		9.100 (7.433, 21.07)	
**Sex**		0.060		0.057
F	32.77 (11.97, —)		— (8.900, —)	
M	11.47 (8.533, 21.20)		8.633 (5.667, 16.17)	
**Line of Therapy**		0.4		0.14
First	16.83 (10.40, 28.20)		14.00 (8.633, —)	
Second or More	11.47 (8.733, —)		6.000 (3.167, —)	
**Race/Ethnicity**		**0.012**		**0.018**
American Indian or Alaska tive	3.033 (—, —)		— (—, —)	
Asian	12.87 (3.767, —)		2.300 (1.167, —)	
Black	— (28.20, —)		— (2.433, —)	
White	11.70 (9.133, 24.83)		9.100 (7.433, —)	
**Child-Pugh Score**		**<0.001**		**0.008**
5	29.27 (24.83, —)		— (12.70, —)	
6	16.90 (11.03, —)		8.667 (4.100, —)	
7	9.533 (3.367, —)		8.633 (3.867, —)	
8	8.333 (5.133, —)		3.167 (1.900, —)	
9	3.033 (2.267, —)		6.000 (0.6000, —)	
10	— (—, —)		4.433 (—, —)	

**Child-Pugh Class**		**<0.001**		**0.013**
A	21.20 (15.10, —)		14.57 (9.100, —)	
B	5.267 (3.233, 11.70)		6.000 (2.633, 21.07)	
C	— (—, —)		4.433 (—, —)	
**BCLC Stage**		0.14		0.13
A	— (21.20, —)		16.33 (9.033, —)	
B	11.97 (5.133, —)		7.067 (4.100, 21.07)	
C	11.47 (9.133, 29.27)		12.70 (6.900, —)	
**ECOG Performance Status**		0.3		0.3
0	11.70 (8.533, —)		14.00 (6.900, —)	
1	16.90 (11.47, 29.27)		9.867 (8.633, —)	
2	8.333 (2.800, —)		3.867 (1.600, —)	
3	5.267 (—, —)		2.433 (—, —)	
**HCC_cause**		0.8		0.2
HBV	8.533 (3.700, —)		1.600 (1.167, —)	
HCV	15.30 (11.47, —)		16.17 (5.667, —)	
Non-viral	11.47 (8.333, —)		9.100 (8.100, —)	
**Prior Locoregional Therapy**		0.9		0.2
No	11.47 (7.367, —)		— (4.833, —)	
Yes	15.10 (10.57, 41.50)		9.033 (7.067, 16.17)	
**Prior Systemic Therapy**		0.4		0.14
No	16.83 (10.40, 28.20)		14.00 (8.633, —)	
Yes	11.47 (8.733, —)		6.000 (3.167, —)	

**Prior Surgery**		0.4		**<0.001**
No	15.10 (10.57, 25.97)		9.867 (8.100, 32.67)	
Yes	3.817 (3.767, —)		0.5833 (0.3667, —)	
**Locoregional Therapy After Atezo Bev therapy**		**0.043**		0.8
No	10.40 (5.267, 28.20)		9.867 (7.067, —)	
Yes	21.20 (16.83, —)		9.100 (6.000, —)	
**Systemic Therapy After Atezo Bev therapy**		0.8		**0.013**
No	11.47 (8.733, —)		14.57 (8.900, —)	
Yes	16.90 (11.70, —)		6.983 (3.867, 16.17)	
**Surgery After Atezo Bev therapy**		0.3		0.6
No	11.70 (9.533, 25.97)		9.100 (7.067, 32.67)	
Yes	26.98 (20.20, —)		16.17 (7.433, —)	

[1] Log-rank test

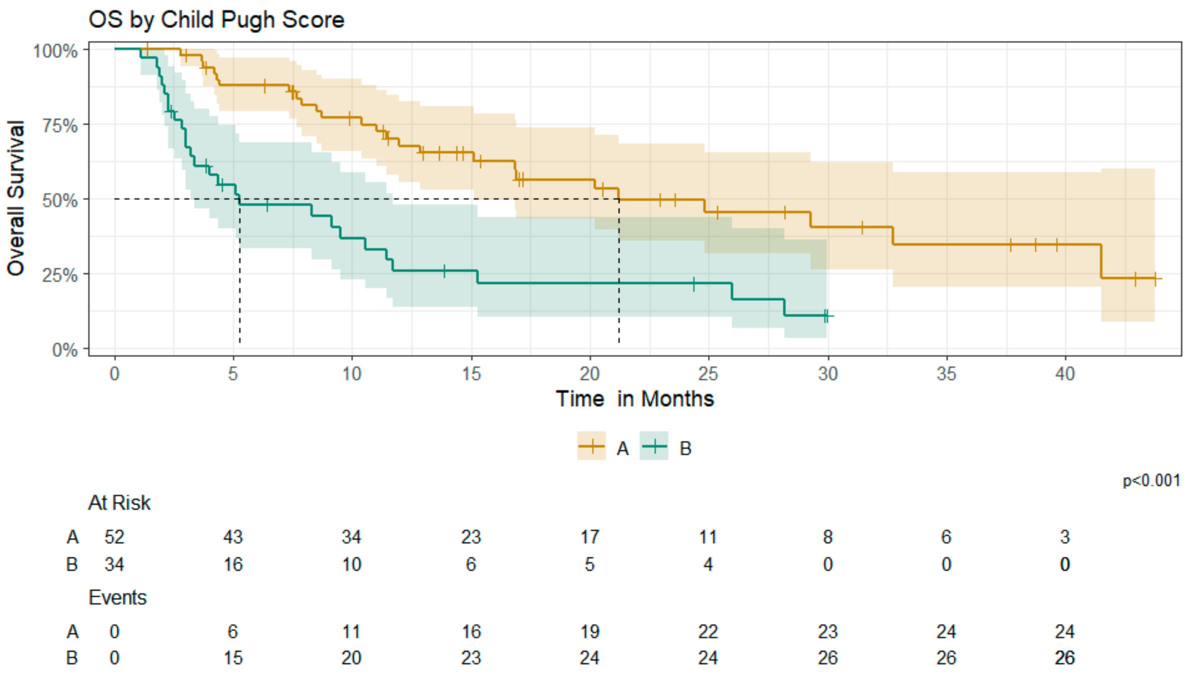

Supplementary Figure S1. Kaplan–Meier OS curves stratified by Child–Pugh score

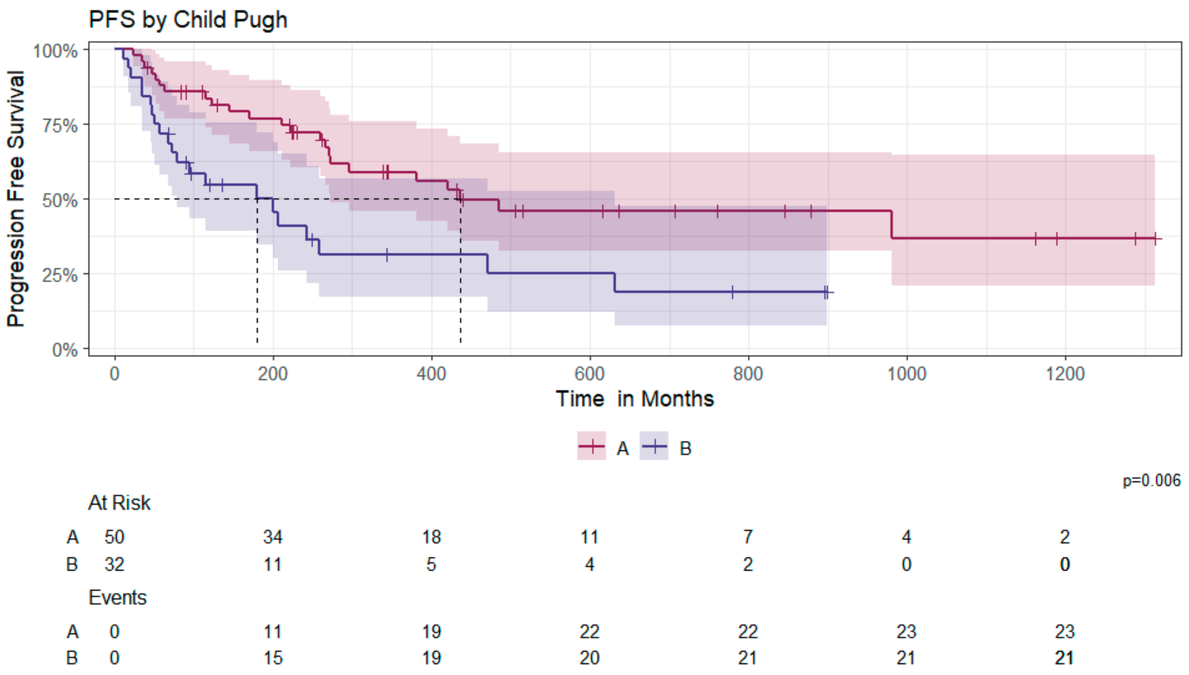

Supplementary Figure S2. Kaplan–Meier PFS curves stratified by Child–Pugh score

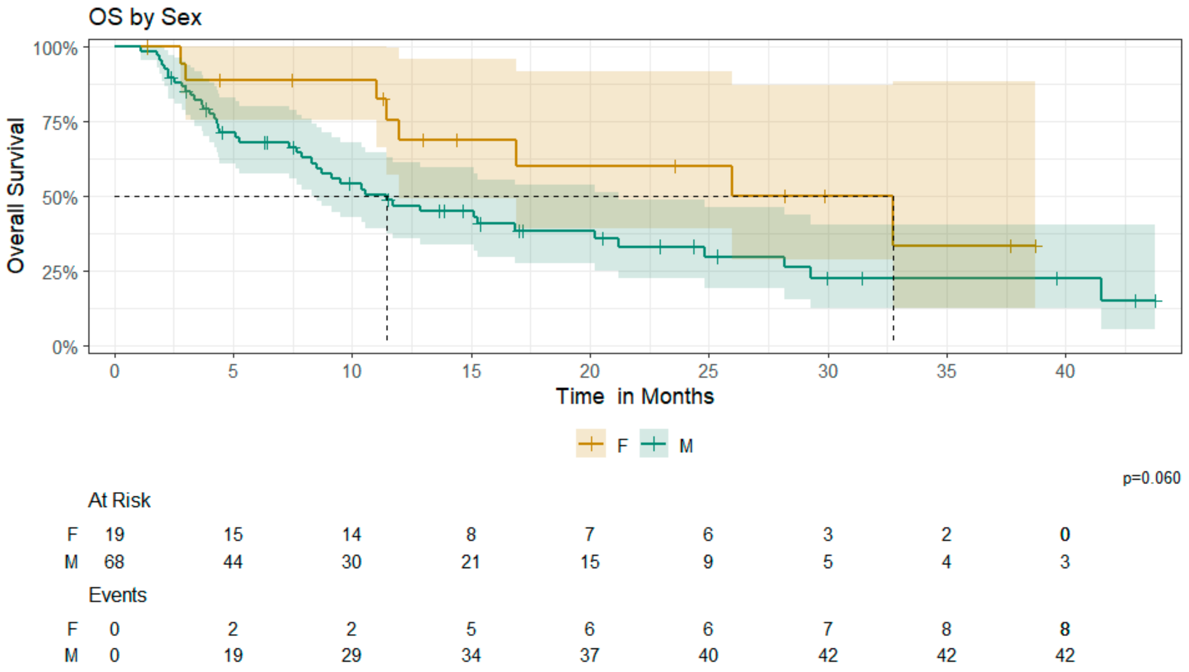

Supplementary Figure S3. Kaplan–Meier OS curves stratified by sex (male vs. female).

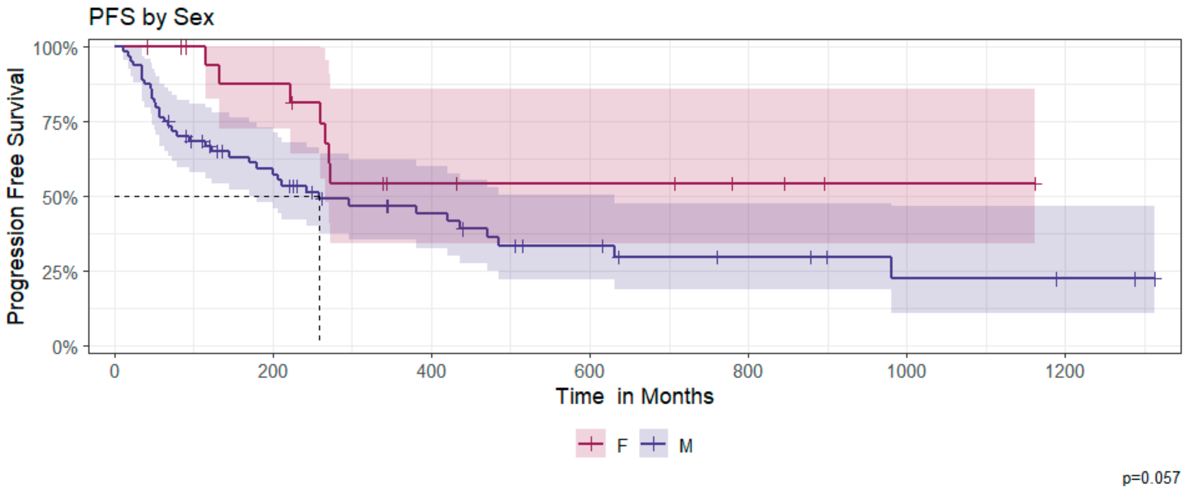

At Risk

F	19	14	6	5	3	1	0
M	64	31	17	10	6	3	2

Events

F	0	2	7	7	7	7	7
M	0	25	32	36	37	38	38

Supplementary Figure S4. Kaplan–Meier PFS curves stratified by sex (male vs. female).

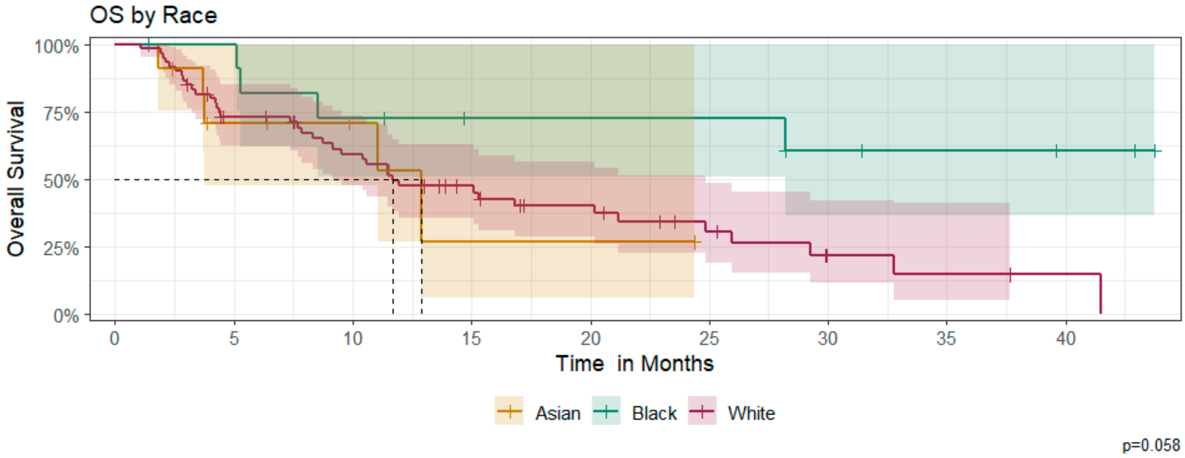

At Risk

Asian	11	6	4	1	1	0	0	0	0
Black	12	11	8	6	6	6	4	3	2
White	60	40	30	20	14	8	3	2	1

Events

Asian	0	3	3	5	5	5	5	5	5
Black	0	0	3	3	3	3	4	4	4
White	0	16	23	29	32	35	37	38	38

Supplementary Figure S5. Kaplan–Meier PFS curves stratified by race.

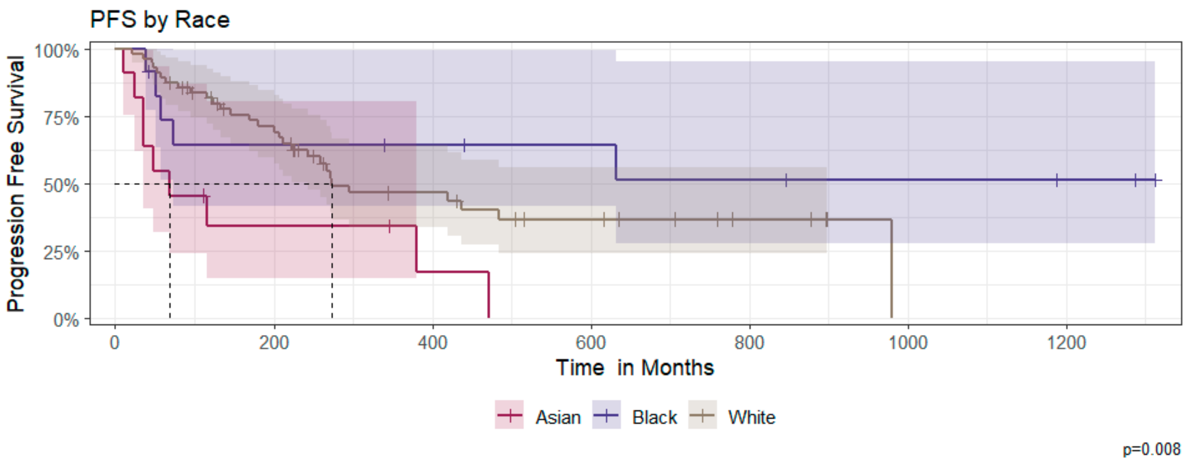

At Risk

Asian	11	3	1	0	0	0	0
Black	12	7	6	5	4	3	2
White	56	33	15	9	4	0	0

Events

Asian	0	7	8	9	9	9	9
Black	0	4	4	4	5	5	5
White	0	15	25	28	28	29	29

Supplementary Figure S6. Kaplan–Meier OS curves stratified by race

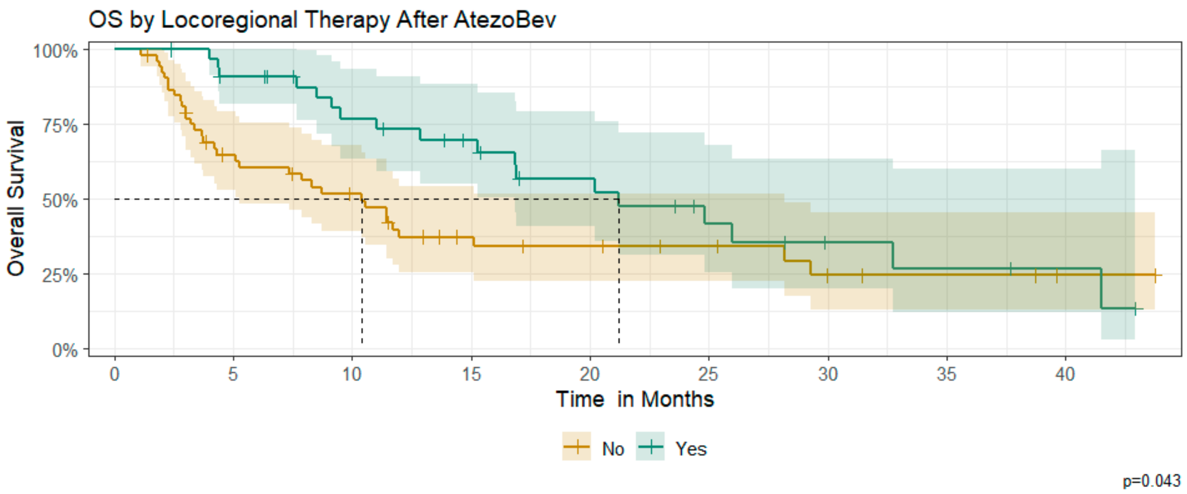

At Risk

No	53	30	22	12	10	8	4	3	1
Yes	34	29	22	17	12	7	4	3	2

Events

No	0	18	24	30	31	31	33	33	33
Yes	0	3	7	9	12	15	16	17	17

Supplementary Figure S7. OS by receipt of locoregional therapy (LRT).

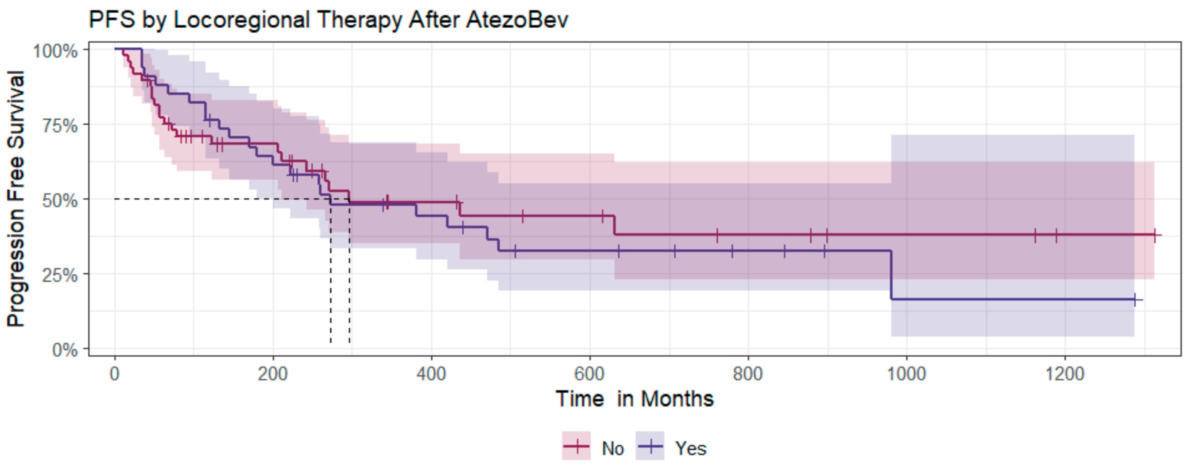

p=0.8

At Risk

	0	200	400	600	800	1000	1200
No	49	24	11	8	5	3	1
Yes	34	21	12	7	4	1	1

Events

	0	200	400	600	800	1000	1200
No	0	15	21	22	23	23	23
Yes	0	12	18	21	21	22	22

Supplementary Figure S8. PFS by receipt of locoregional therapy (LRT).

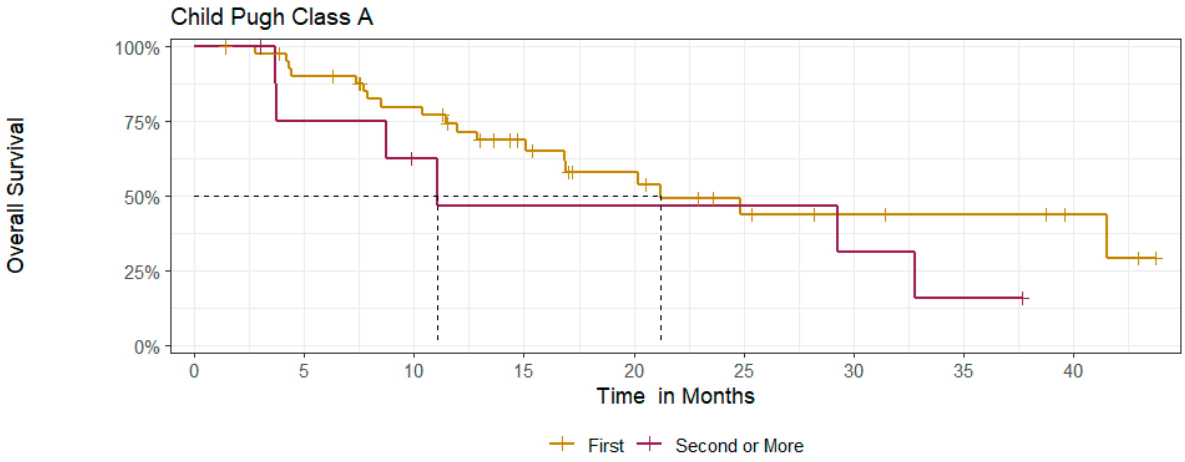

p=0.2

At Risk

	0	5	10	15	20	25	30	35	40
First	43	37	30	20	14	8	6	5	3
Second or More	9	6	4	3	3	3	2	1	0

Events

	0	5	10	15	20	25	30	35	40
First	0	4	8	12	15	18	18	18	18
Second or More	0	2	3	4	4	4	5	6	6

Supplementary Figure S9. OS in Child–Pugh Class A patients by line of therapy.

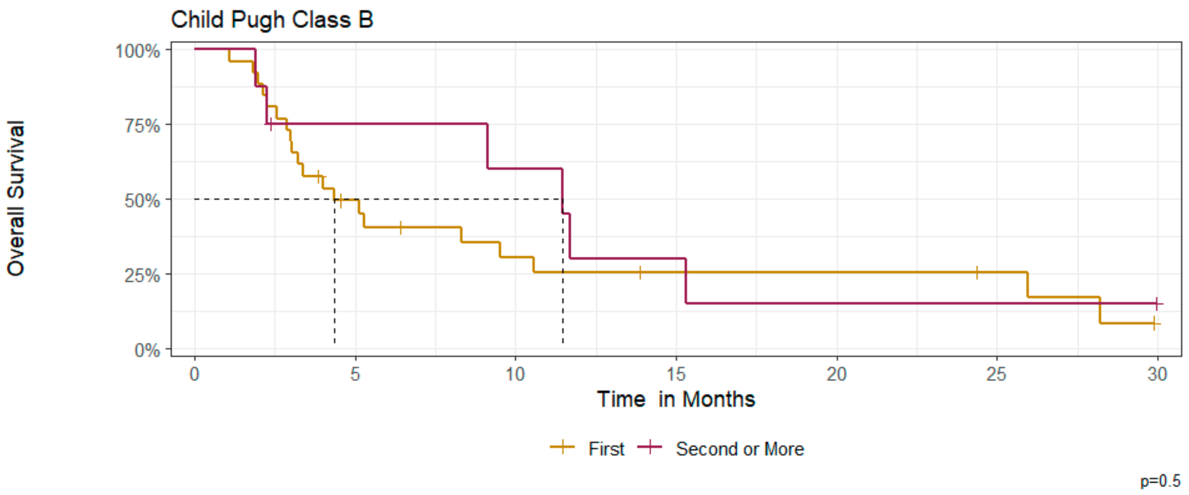

	0	5	10	15	20	25	30
**At Risk**							
First	26	11	6	4	4	3	0
Second or More	8	5	4	2	1	1	0
**Events**							
First	0	13	17	18	18	18	20
Second or More	0	2	3	5	6	6	6

Supplementary Figure S10. OS in Child–Pugh Class B patients by line of therapy.
